# Location and Dynamics of Nymphaeol A in a Complex Membrane

**DOI:** 10.3390/membranes15060163

**Published:** 2025-05-28

**Authors:** José Villalaín

**Affiliations:** Institute of Research, Development, and Innovation in Healthcare Biotechnology (IDiBE), Universidad “Miguel Hernández”, E-03202 Elche-Alicante, Spain; jvillalain@umh.es; Tel.: +34-648891404

**Keywords:** Nymphaeol, antioxidant, molecular dynamics, membrane

## Abstract

Nymphaeol A (NYA) is a tetrahydroxyflavanone anchored to a hydrophobic geranyl group, isolated from different sources and a component of propolis, a complex mixture produced by honeybees and used since ancient times as a healthy drug. This complex exhibits significant antioxidant, antifungal, antibacterial, antiviral, anticancer and antimicrobial properties and NYA is one of its main components. NYA is a lipophilic molecule with two domains, one polar and one hydrophobic. NYA can be inserted into membranes, and its membrane properties depend not only on its location but also on the membrane’s lipid composition. This work uses molecular dynamics to obtain the dynamics, orientation, location and interactions of NYA in a complex biomembrane. This work shows that in an aqueous solution, NYA forms high-order aggregates where the molecules are joined together by the hydrophobic chain. In the presence of a membrane but initially located in the aqueous media, NYA is capable of inserting itself spontaneously into the membrane. Inside the membrane, NYA can be found in the monomeric form, as well as forming aggregates, tending to remain in its most extended conformation. NYA moves along the x-, y- and z-axes, with the movement along the z-axis larger than that of the membrane’s lipids. NYA forms an approximate angle of 35° perpendicular with respect to the membrane and is inserted between the phospholipid hydrocarbon chains, slightly increasing membrane fluidity. Furthermore, NYA prefers POPC and PSM but not POPE or CHOL. NYA’s location and movement within the membrane should be well-suited for its potent bioactivity.

## 1. Introduction

**Figure 1:**
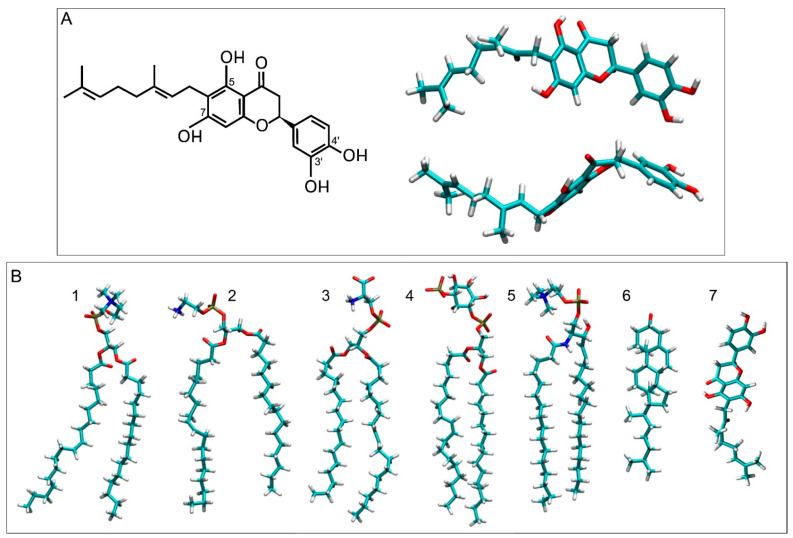
(**A**) Chemical and molecular structures of Nymphaeol A (NYA) and (**B**) the molecular structures of the lipids studied in this work, i.e., POPC (1), POPE (2), POPS (3), PI-3P (4), PSM (5), CHOL (6) and NYA (7), to compare molecular sizes. The molecules are shown in licorice representation.

Nymphaeol A (6-geranyl-3′,4′,5,7-tetrahydroxyflavanone) is a tetrahydroxyflavanone substituted by hydroxy groups at positions 5, 7, 3′ and 4′ with a geranyl group at position 6 (Figure 1A). It has been isolated from different sources, such as *Macaranga tanarius*, and is one of the main components of propolis, a natural product produced by honeybees [1,2,3,4,5,6,7,8,9,10,11,12,13,14,15,16,17]. Propolis is composed of a complex mixture of resins, waxes, essential oils and pollen and has been used since ancient times as a potent drug. It exhibits significant antioxidant, antifungal, antibacterial, antiviral, anticancer and antimicrobial properties. Its phytochemical content varies depending on its botanical and geographical origin, which influences its bioactivity.

Nymphaeol A consists of two well-differentiated parts: a tetrahydroxyflavanone and a geranyl chain. The tetrahydroxyflavanone is a relatively polar domain, whereas the geranyl chain is hydrophobic; however, the whole molecule is very lipophilic (xLogP3 of 5.8). Interestingly, the geranyl group plays an important role in exhibiting antioxidant activity [16]. It is already known that to counteract the existence of free radicals, organisms have different antioxidant defense mechanisms which can be either from exogenous or endogenous origin [18,19,20,21,22,23]; however, antioxidants are the preferred mechanism to inhibit the so-called reactive oxygen species (ROS)’s formation and/or propagation [18,19,24]. High ROS levels lead to oxidative stress, which gives place to neurodegeneration, premature aging and many other illnesses [25]. Since NYA has a great hydrophobic propensity, it could be inserted into a membrane, where it would be capable of quenching and/or scavenging reactive oxygen or nitrogen species at both the surface and interior parts of the membrane. Furthermore, NYA’s position in the membrane could depend on the membrane’s lipid composition and it could have different membrane locations. Because of this, it is important to study its location and interactions in a complex biomembrane.

Understanding how NYA acts is imperative to determine its location, orientation and dynamics inside a membrane and at the same time know its possible effects on the membrane, which could be accountable for its valuable properties on cellular health. Molecular dynamics is suitable to obtain useful data on the structure, dynamics, location and interaction of bioactive molecules within biomembranes [26,27] and it is used in this work to obtain the dynamics, orientation, location and interactions of NYA in a complex biomembrane [28,29] (Table 1). This work shows that NYA molecules in aqueous solution form high-order aggregates where the molecules are joined together by the hydrophobic chain. However, NYA molecules spontaneously insert into the membrane and are finely inserted between the phospholipid hydrocarbon chains, slightly increasing their fluidity. NYA’s location and movement within the membrane is therefore very well-suited for its potent antioxidant activity.

## 2. Materials and Methods

### 2.1. Molecular Dynamics Simulation

Unrestrained all-atom molecular dynamics was performed using NAMD 2.14 [30] and the CHARMM36 protein and lipid force fields [31,32,33]. All molecular dynamics parameters have been previously described [28,29]. The TIP3P model was used for water [34]. The membrane systems were minimized for 150,000 steps to remove bad atomic contacts and then equilibrated for 10 ns. The temperature was 310 K (Table 1).

### 2.2. Molecular Dynamics Specifications

The membranes were obtained using Charmm-Gui (http://www.charmm-gui.org (accessed on 3 January 2025) [35]). All systems contained NaCl at physiological conditions, i.e., a concentration of 150 mM and an excess of water [36] in a neutral environment (Table 1) [28,37].

**Figure 2:**
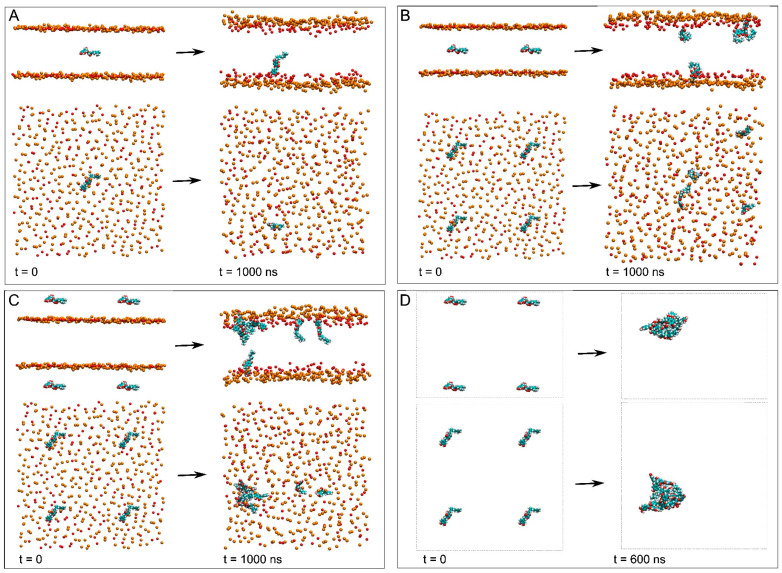
Apical and lateral views of the initial, t = 0 ns, and final, t = 1000/600 ns, dispositions of (**A**) system 1, (**B**) system 2, (**C**) system 3 and (**D**) system 4. The NYA molecules are presented in VDW representation, whereas the phosphate atoms of the phospholipids, defining the upper and lower boundaries of the membrane, and the oxygen atoms of CHOL are represented in VDW and orange and red colors, respectively. The water and lipid molecules and the chloride and sodium ions have been removed for clarity.

The composition of the membrane resembles a general plasma membrane (Table 1) [38,39]. It contained 144 molecules of 1-palmitoyl-2-oleoyl-sn-glycero-3-phosphocholine (POPC), 86 molecules of 1-palmitoyl-2-oleoyl-sn-glycero-3-phosphoethanolamine (POPE), 32 molecules of 1-palmitoyl-2-oleoyl-sn-glycero-3-phosphoserine (POPS), 28 of 1-palmitoyl-2-oleoyl-sn-glycero-3-phosphoinositol-3-phosphorous (PI-3P), 60 molecules of N-stearoyl-D-erythro-sphingosylphosphorylcholine (PSM) and 150 of cholesterol (CHOL) (Table 1) [38,39]. The relative molar percentage of the lipids was 28.8%, 17.2%, 6.4%, 5.6%, 12% and 30% for POPC, POPE, POPS, PI-3P, PSM and CHOL, respectively (Table 1) [38,39]. Three different membrane systems were studied: a membrane system containing only one NYA molecule, system 1 (Figure 2A), a membrane system containing four NYA molecules, system 2 (Figure 2B) and a membrane system containing eight NYA molecules, system 3 (Figure 2C). Additionally, system 4 contained eight NYA molecules inside a water box (Figure 2D). These systems are appropriate to study the interaction of small bioactive molecules with lipids in a membrane [28,37]. The number of lipids in the membrane was 500, with 250 in each layer. The lipid chemical structures are shown in Figure 1A,B. Membrane fluidity was amplified using one oleoyl hydrocarbon chain in the phospholipids [28,37]. PSM contained a palmitoyl hydrocarbon chain apart from the sphingosyl one. The original proportions of the complete systems are shown in Table 1. The molecular structure of NYA was obtained from PubChem (https://pubchem.ncbi.nlm.nih.gov/compound/Nymphaeol-A (accessed on 3 January 2025)) and was reviewed, adjusted and minimized using Discovery Studio 4.0 (Accelrys Inc., San Diego, CA, USA). The CHARMM General Force Field stream files were obtained using Charmm-Gui (http://www.charmm-gui.org [35]).

### 2.3. Molecular Dynamics Analysis

The analysis and visualization were conducted using VMD (Visual Molecular Dynamics, Theoretical and Computational Biophysics Group, University of Illinois at Urbana-Champaign) software and plugins [40,41,42,43,44,45,46,47,48,49]. Data were obtained using the VMD “Membplugin” and “Density Profile Tool” plugins [41,44,45,46,47,49]. The order parameter, S_CD_, was defined as SCD=12 3 cos2 θ−1, where θ is the angle between the C-D vector and the bilayer normal and the brackets symbolize an ensemble average. Hydrogen bonds were as defined previously [45,46]. Data uncertainties represent standard deviation unless otherwise stated.

## 3. Results

### 3.1. NYA in Water

Before studying NYA molecules in the membrane and in order to compare their structures, their behavior in an aqueous solution was studied (system 4, Figure 2D, for the initial and final systems of NYA in water, and Table 1). The system contained eight NYA molecules inside a water box, separated at the beginning by about 60 Å in the x–y plane and by about 70 Å in the z plane. The molecules were free to wander in the water solution, so that at the end of the simulation, i.e., 600 ns, all eight molecules formed an aggregate (Figure 2D and Supplementary Figure S1A). The average distances between the carbon atoms at position 4′ and 13 (the last carbon of the geranyl chain) for the last 30 ns are shown in Supplementary Figure S1B. All of them are around 13–14 Å, the global average of the eight NYA molecules being 13.7 ± 1.0 Å (rounded to the first decimal). Since the distance between these two atoms in the NYA’s most extended conformation is about 15.8 Å, it is possible to say that NYA in an aqueous solution remains nearly in its most extended conformation. As was commented above, the NYA molecule could be divided into two distinct parts: a relatively polar part (tetrahydroxyflavanone group) and a hydrophobic part (geranyl group) (Figure 1A). The oligomer structure is shown in Supplementary Figure S1A, where it is possible to see that the molecules are joined together by the hydrophobic part, i.e., the geranyl chain, forming a kind of sphere, where the polar groups tend to be at the surface. It seems that hydrophobic forces are accountable for attraction of different molecules, whereas the polar terminal groups might stabilize the aggregate (Supplementary Figure S1A).

### 3.2. Membrane Systems

Three different membrane systems were studied: a membrane system containing only one NYA molecule, system 1 (Figure 2A), a membrane system containing four NYA molecules, system 2 (Figure 2B), and a membrane system containing eight NYA molecules, system 3 (Figure 2C). The NYA molecules in systems 1 and 2 were located in the center of the membrane at time 0, whereas the NYA molecules in system 3 were located in the center of each one of the water layers outside the membrane [37]. It should be taken into account that they were very diluted systems, since the membrane contained 500 lipids, with 250 in each monolayer. The membrane thickness and lipid areas were checked to assess the equilibration of the systems during the molecular dynamics [50,51]. After ~70 ns, the mean membrane thickness was almost the same for all the systems, being for the last 30 ns nearly indistinguishable, i.e., between 45.8 and 46 Å, as shown in Supplementary Figure S2. These data are comparable to those described previously [52]. The mean molecular areas for all systems and for the last 30 ns of molecular dynamics for POPC, POPE, POPS and PI-3P ranged between 56 and 57 Å^2^, for PSM ranged between 50 and 51 Å^2^, and for CHOL ranged between 28.6 and 28.9 Å^2^. These areas were similar to those formerly reported [44,52,53]. Accordingly, all membranes were equilibrated very early and were in a steady state after ~70 ns of molecular dynamics.

### 3.3. NYA Inside the Membrane

**Figure 3:**
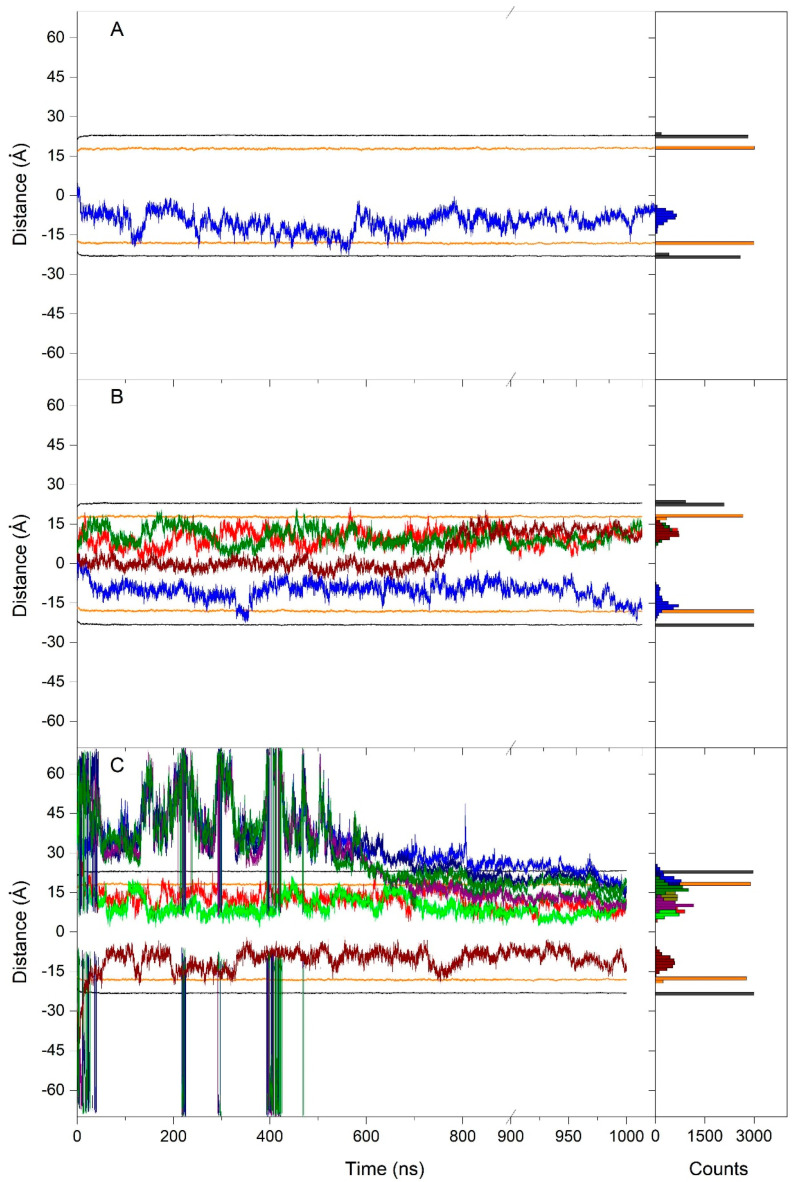
Time variation of the NYA whole-molecule z-axis COM distance pertaining to (**A**) system 1 (one NYA molecule), (**B**) system 2 (four NYA molecules) and (**C**) system 3 (eight NYA molecules). Each NYA molecule is represented by a different color. The corresponding histograms are shown at the right margin (the data are the averages of the last 30 ns of molecular dynamics simulation). The z-axis COM distance of the phosphate atoms of the phospholipids, defining the membrane surface, is depicted in black, whereas the z-axis distance of the oxygen atoms of cholesterol is depicted in orange (upper and lower boundaries).

**Figure 4:**
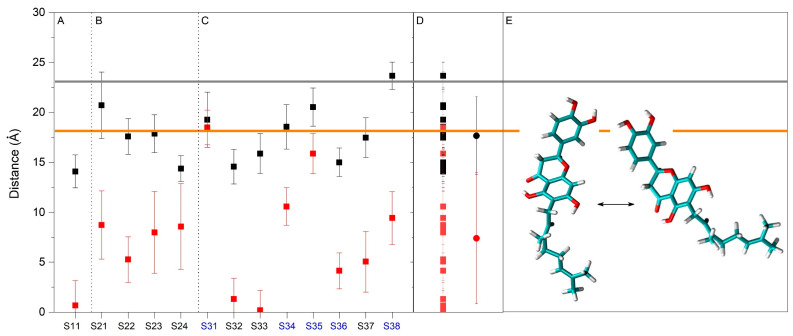
Average z-axis COM distance of the last 30 ns of molecular dynamics simulation of the carbon C4′ atom (■) and the terminal carbon of the geranyl chain (■) pertaining to (**A**) system 1 (one NYA molecule), (**B**) system 2 (four NYA molecules) and (**C**) system 3 (eight NYA molecules). Blue marks in (**C**) denote those NYA molecules in aggregated form inside the membrane. In (**D**), all data are represented at the same time, as well as their average ± SD, whereas in (**E**) the possible location of NYA in the membrane is represented. The z-axis COM distance of the phosphate atoms of the phospholipids, defining the membrane surface, is depicted as a dark grey line, whereas the z-axis distance of the oxygen atoms of cholesterol is depicted as an orange line.

System 1 originally had one molecule of NYA located at the membrane’s central part (Figure 2A). At t = ∞ of the molecular dynamics simulation, i.e., 1000 ns, the NYA molecule proceeded to a location adjacent to the membrane interphase and inserted in between the phospholipid hydrocarbon layer, with the hydrophobic chain pointed towards the middle of the membrane (Figure 2A). Its behavior throughout the molecular dynamics simulation can be observed by studying the whole-molecule z-axis center-of-mass (z direction normal to the bilayer plane, z-COM) (Figure 3A). This can be compared to the phosphate atoms’ z-COMs, which define the membrane surface, and the CHOL oxygen atoms, which define the interphase (Figure 3A). As commented above, at the beginning, the NYA molecule was located in the middle of the membrane; however, with time, it spontaneously positioned itself at the middle of one of the monolayers, this being its average position for the last 30 ns of −7.4 Å ± 1.8 Å (Figure 3A). This location was maintained for nearly all molecular dynamics simulations. It is also possible to observe that its displacement along the z-axis was larger than those of the phosphate atoms of the phospholipids and the oxygen atom of CHOL. However, NYA is a relatively long molecule, its maximum extension being about 15.8 Å (Figure 1B). Because of that, this is interesting, not only knowing its global location but also the location of its extremes, namely the z-COM values of the carbon C4′ atom (−14.1 Å ± 1.6 Å) and C13 atom (−0.7 Å ± 2.4 Å) (Figure 4A). This would imply that the molecule moves in a perpendicular way to the membrane’s surface along the z-axis, with the tetrahydroxyflavanone domain pointing towards the membrane interphase, whereas the geranyl domain points towards the middle of the membrane. This work also analyzed the distance between carbons C4′ and C13 and the data are shown in Supplementary Figure S3A. As shown in the figure, the average distance is 15.0 ± 1.7 Å for the last 30 ns of the simulation. Since the distance between these two atoms in the most extended conformation is about 15.8 Å, the NYA molecule inside the membrane is near its most extended conformation.

System 2 originally had four molecules of NYA located at the membrane’s central part (Figure 2B). At t = ∞ of the molecular dynamics simulation, i.e., 1000 ns, the NYA molecules proceeded to a location close to the membrane interphase and inserted in between the phospholipid hydrocarbon layer, with the hydrophobic chain pointed towards the middle of the membrane in a similar way to the NYA molecule in system 1 (Figure 2A). Although the NYA molecules were located in the middle of the membrane, with time, they spontaneously positioned themselves at the middle of the monolayers, their average positions for the last 30 ns being −15.1 Å ± 2.8 Å, 12.0 Å ± 1.6 Å, 12.0 Å ± 2.2 Å and 11.8 Å ± 1.8 Å (Figure 3A). As observed in the Figure, of the four molecules, three arranged themselves rapidly in the middle of each of the monolayers; the remainder remained in the middle of the membrane until about 800 ns, and from that time onwards it was located in the middle of one of the monolayers (Figure 3A). Similarly to the NYA molecule in system 1, their displacement along the z-axis was larger than those of the phosphate atoms of the phospholipids and the oxygen atom of CHOL. The locations of their extremes, namely the z-COM values of the carbon C4′ and C13, were −20.7 Å ± 3.3 Å and −8.8 Å ± 3.4 Å, 17.6 Å ± 1.8 Å and 5.3 Å ± 2.3 Å, 17.9 Å ± 1.9 Å and 8.0 Å ± 4.1 Å and finally 14.4 Å ± 1.3 Å and 8.6 Å ± 4.3 Å, respectively (Figure 4B). This would imply that the molecules are capable of moving in a perpendicular way to the membrane’s surface along the z-axis, with the tetrahydroxyflavanone domain pointed towards the membrane interphase, whereas the geranyl domain points towards the middle of the membrane. This work also analyzed the distance between the carbon C4′ and C13 atoms and the terminal carbon of the geranyl chain, and the data are shown in Supplementary Figure S3B. As shown in the figure, the average distances were 14.6 Å ± 2.1 Å, 13.9 Å ± 2.4 Å, 12.6 Å ± 2.3 Å and 14.3 Å ± 1.0 Å, respectively, for the last 30 ns of the simulation. Similarly to the NYA molecule in system 1, the NYA molecules in system 2 are close to their most extended conformation.

System 3 originally had eight molecules of NYA positioned in the middle of each water layer (Figure 2C). At t = ∞ of the molecular dynamics simulation, i.e., 1000 ns, the NYA molecules proceeded to a position close to the membrane interphase and inserted in between the phospholipid hydrocarbon layer, with the hydrophobic chains pointed towards the middle of the membrane in a similar way to the NYA molecules in system 1 and system 2 (Figure 2A,B). Although the NYA molecules were located in the water layers, at different times they spontaneously positioned themselves at the middle of the monolayers, remaining there until the end of the simulation. Their average positions for the last 30 ns were 19.7 Å ± 2.2 Å, 8.0 Å ± 1.6 Å, 8.4 Å ± 2.0 Å, 13.3 Å ± 1.9 Å, 17.8 Å ± 2.1 Å, 10.4 Å ± 1.4 Å, −11.4 Å ± 2.1 Å and 16.9 Å ± 1.5 Å, respectively (Figure 3A). Three of the NYA molecules did insert into the membrane very quickly (at 16, 18 and 20 ns, respectively), while the remaining five did so later and at different times (Figure 3A). These NYA molecules interacted with and inserted into the membrane at about 610, 620, 630, 714 and 825 ns (Figure 3A). Similarly to the NYA molecules in the other systems, once inserted into the membrane, their displacement along the z-axis was larger than those of the phosphate atoms of the phospholipids and the oxygen atom of CHOL. The locations of their extremes, namely the z-COM values of the carbon C4′ and the terminal carbon of the geranyl chain, were 19.3 Å ± 2.8 Å and 18.5 Å ± 1.7 Å, 14.6 Å ± 1.7 Å and 1.3 Å ± 2.1 Å, 15.9 Å ± 2.0 Å and 0.2 Å ± 2.0 Å, 18.5 Å ± 2.2 Å and 10.6 Å ± 1.9 Å, 20.5 Å ± 1.9 Å and 15.9 Å ± 2.0 Å, 15.0 Å ± 1.4 Å and 4.1 Å ± 1.8 Å, −17.5 Å ± 2.0 Å and −5.0 Å ± 3.0 Å and finally 23.6 Å ± 1.4 Å and 9.4 Å ± 2.7 Å, respectively (Figure 4C). It is highlighted that the three NYA molecules that were rapidly inserted into the membrane remained in a monomeric state for the entire simulation, while the NYA molecules that were inserted later joined together, forming a 5-unit oligomer at the membrane interface. The five monomers formed an oligomer in solution beforehand, and this oligomer subsequently inserted into the membrane through the geranyl chains of two NYA molecules (about 620 ns, Figure 3C). We have also analyzed the distance between carbon C4′ and C13 atoms and the data are shown in Supplementary Figure S3C. As shown in the figure, the average distances were 15.5 Å ± 0.7 Å, 14.7 Å ± 1.7 Å, 16.2 Å ± 0.9 Å, 14.1 Å ± 2.0 Å, 15.3 Å ± 0.9 Å, 14.7 Å ± 1.4 Å, 15.6 Å ± 1.1 Å and 15.0 Å ± 1.9 Å, respectively, for the last 30 ns of the simulation. Similarly to the NYA molecules in systems 1 and 2, the NYA molecules are close to their most extended conformation. In a similar way to the oligomer formed in water, the molecules in the oligomer inside the membrane were joined together by the hydrophobic part, i.e., the geranyl chain.

Summarizing the results, it is possible to say that NYA inserts spontaneously inside the membrane and its most probable location is in the middle of the membrane monolayer (the global average location of NYA inside the membrane is 14.7 Å ± 1.4 Å, Supplementary Figure S3D). The NYA molecules were capable of moving in a perpendicular way to the membrane’s surface along the z-axis, with the tetrahydroxyflavanone domain pointed towards the membrane interphase, whereas the geranyl domain pointed towards the middle of the membrane (Supplementary Figure S3D,E). Interestingly, NYA is always close to its most extended conformation and it is possible for the molecule to be a monomer or in an aggregated form inside the membrane.

### 3.4. Angles Formed Between NYA and the Membrane

As commented above, the NYA molecules insert well in between the hydrocarbon chains of the phospholipids, remaining in their nearly most extended conformation, 14–15 Å. Using the z-COM data of the extremes of the molecule, it is possible to infer the relative angle of NYA in the membrane (Figure 4E). Nevertheless, this work obtained the average of the angle formed by the whole molecules in the membrane for the last 30 ns of molecular dynamics, and the data can be observed in Supplementary Figure S4 (average angles formed by carbons C4′ and C13, the last one of the geranyl chain, for the NYA molecules in the monomeric form with respect to the membrane’s surface). The average angles were relatively similar for all of the NYA molecules in the monomeric form, i.e., 24.2° ± 9.3°, 43.6° ± 17.1°, 46.3° ± 12.7°, 27.5° ± 14.3°, 75.0° ± 6.9°, 24.9° ± 13.9°, 38.3° ± 12.6° and 11.3° ± 6.7° (Supplementary Figure S4). The global average angle was 36.4° ± 19.4° (Supplementary Figure S4). These data are in agreement with the data inferred by the z-COM data commented above. Although the NYA molecules present different conformations, their preferred disposition is approximately the same for all of them and forms an approximate angle of 36° with respect to the membrane.

### 3.5. Lipid Density Profiles

The lipid mass density profiles are shown in Supplementary Figure S6A,B for system 1, Supplementary Figure S6C,D for system 2 and Supplementary Figure S6E,F for system 3. The lipid mass density profiles were essentially symmetric between the two membrane layers, representing the comparable behavior of the lipids. This is the expected comportment, since all the systems were very diluted. It can be also observed in the figures the positions of the NYA molecules with respect to the lipids in the membrane, where all of them reside relatively at the same depth in the membrane. We should remember that the NYA molecules are relatively narrow and long molecules with two distinct moieties; the hydrophobic tail and the tetrahydroxyflavanone moiety. As observed in Supplementary Figure S5, the tetrahydroxyflavanone moiety tends to be between the oxygen atom of CHOL and the first carbons of the phospholipid hydrocarbon chain, whereas the geranyl chains tends to be located between the middle of the phospholipid hydrocarbon chain and the membrane middle. These data also show that the locations of the NYA molecules in the membrane are very similar between the different molecules and relatively well defined. This should be related to the geometry and structure of the molecules and their location should favor their bioactivity.

### 3.6. Phospholipid Hydrocarbon Chain Order in the Presence of NYA

Bioactive molecules inside a membrane can alter the phospholipid hydrocarbon chain order. As such, this work explored the consequence of the NYA molecules obtaining the deuterium order parameter, S_CD_, for the phospholipids in systems 1, 2 and 3 (NYA molecules in the monomeric form). The S_CD_ values are shown in Supplementary Figure S6. The S_CD_ data of the hydrocarbon chains of the bulk phospholipids were comparable with the profiles previously observed for biomembranes for both experimental and simulated data [33,54,55,56]. For the phospholipid hydrocarbon chains near the NYA molecules, globally speaking, there were some differences in the S_CD_ profiles: the S_CD_ values were slightly higher in the first half of the chains in the presence of the NYA molecules, but in the second half the S_CD_ values were slightly lower. This is in accordance with the data shown above: the tetrahydroxyflavanone moiety tends to be between the oxygen atom of CHOL and the first carbons of the phospholipid hydrocarbon chain, whereas the geranyl chain tends to be located between the middle of the phospholipid hydrocarbon chain and the membrane’s middle. To know the global effect these bioactive molecules have on lipid fluidity, this work detracts the S_CD_ values of the bulk lipids minus the S_CD_ values of the surrounding ones: if the result is negative, the molecule has an ordering effect; if positive, it has a disordering effect (Supplementary Figure S6K). These data show that NYA molecules are ordered in the first half of the phospholipid hydrocarbon chains, where the tetrahydroxyflavanone tends to be located (decrease of membrane fluidity), but disordered in the second half, where the geranyl chain tends to be located (increase of membrane fluidity). However, they do not affect dramatically the hydrocarbon chains’ anisotropy.

### 3.7. Lipid Molecules near NYA

**Figure 5:**
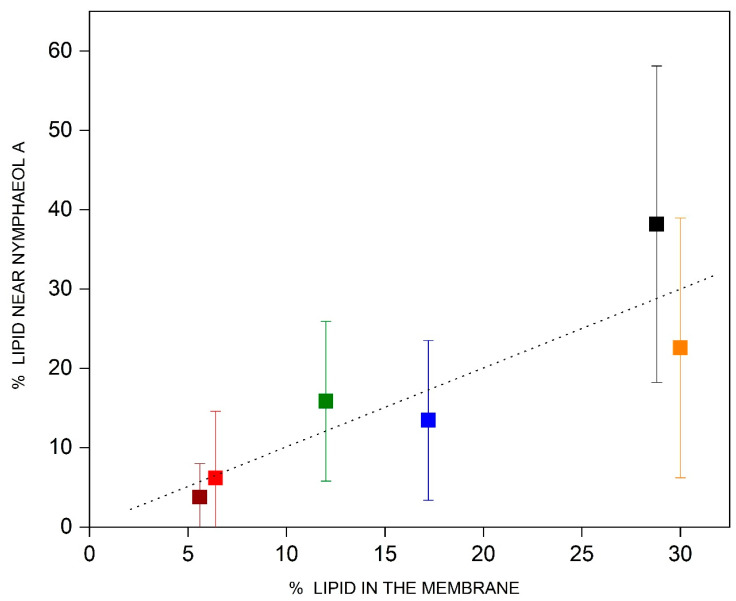
Percentage of the number of lipid molecules in the membrane versus observed number of lipid molecules at a distance of 6 Å from the NYA molecules: POPC (-■-), POPE (-■-), POPS (-■-), PI-3P (-■-), PSM (-■-) and CHOL (-■-). The dotted line represents an identical observed versus expected number of lipid molecules. The analysis was carried out for the last 30 ns of simulation.

This work obtained the type and number of lipid molecules at a distance of 6 Å from NYA and compared them with the bulk quantities, i.e., analyzed if any lipid type was overrepresented or diminished near the bioactive molecules (average over the last 30 ns of the molecular dynamics and for systems 1, 2 and 3, Figure 5). The percentages of POPC and PSM were relatively higher than the bulk percentages (about 38% vs. 28.8% and about 16% vs. 12%, respectively), whereas the percentages of POPE and CHOL were lower than the bulk ones (about 13% vs. 17.2% and about 22% vs. 30%, respectively) (Figure 5). The percentages of POPS and PI-P3 were relatively similar to the corresponding bulk percentages (6% vs. 6.4% and 4% vs. 5.6%, respectively). In general, it can be said that NYA would prefer POPC and PSM but not POPE or CHOL.

### 3.8. Diffusion Coefficients

This work measured the diffusion coefficients of all the bioactive and lipid molecules in all the systems and the data are shown in Supplementary Figure S7. As observed in Supplementary Figure S7, the lipid diffusion coefficients on the x- and y-axes range from about 3.4 Å^2^/ns (POPC) to 8.8 Å^2^/ns (PI-P3), with the values of the other lipids in between. The average x–y diffusion coefficient of the lipids is 5.7 ± 2.8 Å^2^/ns. These values can be compared with the x–y diffusion coefficient of NYA, which is 0.5 ± 0.3 Å^2^/ns. Therefore, the x–y diffusion coefficient of NYA is much lower than the x–y diffusion coefficient of the lipids. The lipid diffusion coefficients on the z-axis range from about 0.08 Å^2^/ns (POPE) to 0.05 Å^2^/ns (CHOL), with the values of the other lipids in between. The average z diffusion coefficient of the lipids is 0.06 ± 0.02 Å^2^/ns. These values can be compared with the z-axis diffusion coefficient of NYA which is 0.2 ± 0.1 Å^2^/ns, i.e., about three times larger than the lipids. Therefore, the diffusion of NYA in the membrane x–y plane is lower than the diffusion of the lipids and the diffusion of NYA in the z direction is larger than the diffusion of the lipids (Supplementary Figure S7).

### 3.9. Hydrogen Bonds

This work also obtained the mean number of hydrogen bonds formed between the NYA molecules and the membrane lipids for the last 30 ns of the simulation (Supplementary Figure S8). POPC and PSM were the lipids which showed a relatively major proportion of hydrogen bonds (about 1 and 2, respectively); the other lipids did not show the presence of any hydrogen bonds at all (POPE, POPS, PI-3P and CHOL). The data shown are in accordance with the above-commented data that NYA would prefer POPC and PSM rather than the other lipids. Since the relative molar percentage of POPC and PSM in the membrane is about 40% of the total lipids, NYA should be relatively free to move within the membrane.

## 4. Discussion

**Figure 6:**
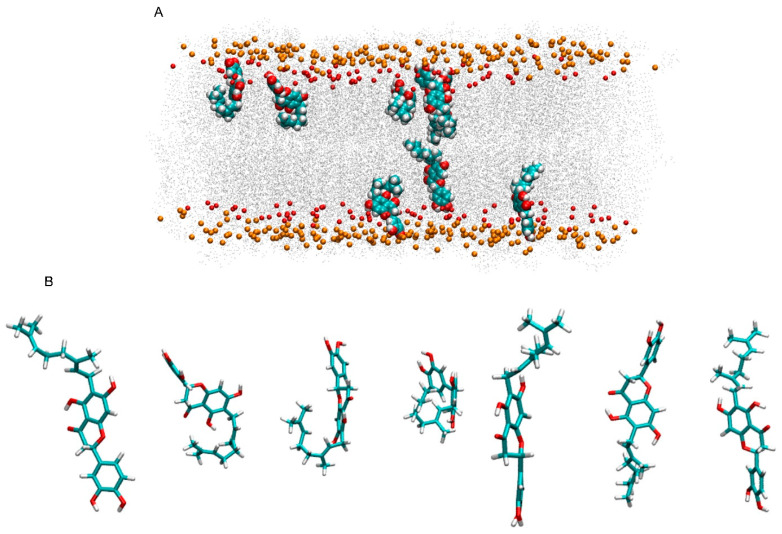
(**A**) Representation of the location of NYA molecules in the membrane (monomeric form, final frame of the molecular dynamics simulation). The NYA molecules are presented in VDW representation, as well as the phosphate atoms of the phospholipids (orange, size reduced by half), defining the upper and lower boundaries of the membrane, and the oxygen atoms of cholesterol (red, size reduced by half). For clarity, lipid atoms are represented as grey dots, while water and chloride and sodium ions have been removed. (**B**) NYA molecules in (**A**) highlighting the different conformations observed.

Nymphaeol A is a tetrahydroxyflavanone anchored to a hydrophobic geranyl group, isolated from different sources and a component of propolis, a complex mixture produced by honeybees and used since ancient times as a healthy drug. Nymphaeol A is a relatively lipophilic molecule, having two domains, one polar and another hydrophobic, and therefore it could insert into a membrane, where it would be capable of quenching and/or scavenging reactive oxygen or nitrogen species. I have used molecular dynamics to obtain the location, orientation, dynamics and interactions of NYA in a complex biomembrane (Figure 6). This work shows that NYA molecules in an aqueous solution form high-order aggregates where the molecules are joined together by the hydrophobic chain. In the presence of a membrane but initially located in aqueous media, NYA molecules were capable of inserting themselves into the membrane; inside the membrane, NYA can be found in its monomeric form, as well as forming aggregates, tending to remain in its most extended conformation. Interestingly, NYA can form oligomers in aqueous solution, but its formation does not preclude the oligomer from inserting spontaneously into the membrane. NYA moves along the x-, y- and z-axes, with the movement along the z-axis larger than that of the membrane lipids, but lower in the x–y plane. In this way, NYA moves much better perpendicular to the membrane’s surface. Although NYA molecules in the membrane can present different conformations, they tend to form an approximate angle of 36°, perpendicular with respect to the membrane, and be inserted between the phospholipid hydrocarbon chains. Therefore, NYA tends to be in its most extended conformation. In doing so, NYA increases membrane fluidity, but without dramatically affecting its anisotropy. It is interesting to comment that NYA would prefer POPC and PSM but not POPE or CHOL. Regardless of this preference, which is not exclusive, the fact that, on the one hand, fluidity is little affected by NYA and, on the other hand, that it had a certain preference for PSM but not for CHOL indicates there is no evidence that the incorporation of NYA into the membrane can lead to the formation of specific domains. The average location of NYA in the membrane lies in the upper part of the membrane’s hydrocarbon structure, with the tetrahydroxyflavanone domain pointed towards the membrane interphase, whereas the geranyl domain points towards the middle of the membrane. In this way, NYA can reach the membrane interface where the carbonyl groups of phospholipids are located. Since one of the most important bioactive properties of NYA is that it is an antioxidant molecule, its location in the membrane is essential to protect lipids from the harmful effects of reactive oxygen species. NYA’s location and movement within the membrane is therefore well-suited for its bioactivity.

## 5. Conclusions

NYA, a bioactive molecule composed of two characteristic domains, is relatively lipophilic and spontaneously inserts into the biological membrane. Using molecular dynamics, this work has been able to determine that it forms oligomers in an aqueous solution without preventing its insertion into the membrane, it is capable of moving much faster along the z-axis than membrane lipids, it tends to be in its most extended conformation, it slightly increases membrane fluidity, it has a preference for POPC and PSM but not for POPE and CHOL and it is also capable of locating itself at the upper part of each monolayer, reaching the carbonyl groups of phospholipids. The location and movement of NYA within the membrane are well-suited to its bioactivity and essential for protecting lipids from the damaging effects of reactive oxygen species.

## Figures and Tables

**Table 1:** Membrane systems and number of components. The NaCl concentration was 0.15 M. The time to obtain the production trajectories for each one of the systems is also indicated. The total number of lipid molecules was 500, with 250 per monolayer.

Systems		1	2	3	4
Nymphaeol A		1	-	-	-
Nymphaeol A		-	4	-	-
Nymphaeol A		-	-	8	-
Nymphaeol A		-	-	-	8
MD time (ns)		1000	1000	1000	600
POPC	28.8%	144	144	144	-
POPE	17.2%	86	86	86	-
PI-3P	5.6%	28	28	28	-
POPS	6.4%	32	32	32	-
PSM	12%	60	60	60	-
CHOL	30%	150	150	150	-
ATOMS		166,370	166,547	166,234	167,176
H_2_O		36,413	36,413	36,230	55,464
H_2_O/LIPID		72.8	72.8	72.5	-
Na^+^		219	219	219	156
Cl^−^		103	103	103	156
DIMENSIONS x/y/z (Å)		128/129/120	128/129/120	128/129/120	128/129/120

## Data Availability

The original contributions presented in this study are included in the article/Supplementary Materials. Further inquiries can be directed to the corresponding author.

## Supplementary Materials

The following supporting information can be downloaded at: https://www.mdpi.com/article/10.3390/membranes15060163/s1, Figure S1: (A) Structure of the aggregate of NYA molecules at the last frame for system 4 (t = 600 ns). NYA molecules (eight) are shown in licorize representation, whereas the oxygens are shown in VDW representation (hydroxyl, carbonyl and ether oxygens in red, green and orange colours, respectively). (B) Average distance between the carbons C4’ and the last one of the geranyl chain in the aggregate for each one of the NYA molecules as well as their average. Data correspond to the last 30 ns of molecular dynamics simulation; Figure S2: Time variation of membrane thickness for the whole simulation time and the corresponding histograms for the last 30 ns of molecular dynamics simulation. Thickness corresponds to the phosphate atoms of the phospholipids (upper lines) and the oxygen atoms of cholesterol (lower lines) for system 1 (black), system 2 (blue) and system 3 (red); Figure S3: Average distance between carbons C4’ and the last one of the geranyl chain for the NYA molecules pertaining to (A) system 1 (one NYA molecule), (B) system 2 (four NYA molecules), and (C) system 3 (eight NYA molecules). In (D) the average ± SD is shown. Data correspond to the last 30 ns of molecular dynamics simulation; Figure S4: Histograms and average angles (± SD) formed by carbons C4’ and the last one of the geranyl chain for the NYA molecules in monomeric form with respect to the membrane surface. The full symbols correspond to the average angle of each one of the NYA molecules, whereas the empty symbol corresponds to the global average. The data is the average for the last 30 ns of molecular dynamics simulation time; Figure S5: Mass density profiles for the last 30 ns of the molecular dynamics for (A,B) system 1, (C,D) system 2 and (E,F) system 3. (A,C,E) Whole lipid molecules and (B,D,F) phosphorous atoms of phospholipids and the oxygen atoms of CHOL. POPC is shown in black, POPE in blue, POPS in red, PI-3P in olive, PSM in wine and CHOL in orange. The monomeric and aggregated NYA molecules are shown in dotted black and blue colours; Figure S6: Average deuterium order parameter, –S_CD_, calculated for the hydrocarbon chains of the phospholipids. (A,C,E,G) Oleoyl and (B,D,F,H) palmitoyl acyl chains of (A, B) POPC, (C, D) POPE, (E, F) POPS, (G,H) PI-3P, as well as the palmitoyl (I) and sphyngosyl (J) acyl chains of PSM. The data correspond to the bulk phospholipid acyl chains (-■-) and the phospholipid acyl chains within 6 Å of the NYA molecule (-●-). The analysis was carried out for the last 30 ns of simulation. (K) Sum of differences between the -S_CD_ values of the bulk lipids minus the S_CD_ values of the surrounding ones; Figure S7: (A) x-y and (B) z diffusion coefficients (Å^2^/ns) for the NYA molecules in systems 1, 2 and 3 in the monomeric form: POPC (■), POPE (■), POPS (■), PI-3P (■), PSM (■), CHOL (■) and NYA (■). The data is the average for the last 30 ns of molecular dynamics simulation time; Figure S8: Average number of hydrogen bonds between the membrane lipids and NYA: POPC (-■-), POPE (-■-), POPS (-■-), PI-3P (-■-), PSM (-■-) and CHOL (-■-). The analysis was carried out for the last 30 ns of simulation.

## Abbreviations

NYA	6-geranyl-3′,4′,5,7-tetrahydroxyflavanone
CHOL	cholesterol
PI-3P	1-palmitoyl-2-oleoyl-sn-glycero-3-phosphoinositol-3-phosphate
POPC	1-palmitoyl-2-oleoyl-sn-glycero-3-phosphocholine
POPE	1-palmitoyl-2-oleoyl-sn-glycero-3-phosphoethanolamine
POPS	1-palmitoyl-2-oleoyl-sn-glycero-3-phospho-L-serine
PSM	N-palmitoyl-D-erythro-sphingosylphosphorylcholine
z-COM	z-axis center-of-mass (z direction normal to the bilayer plane)
