# Supplementary material for: Location and Dynamics of Nymphaeol A in a Complex Membrane
Source: Membranes (Basel). 2025 May 28;15(6):163. doi: 10.3390/membranes15060163 (PMC12195524; doi:10.3390/membranes15060163)
Supplement: Supplementary file 1 [file membranes-15-00163-s001.zip › membranes-3657967-supplementary.pdf]

SUPPLEMENTARY MATERIAL

Supplementary Figure S1.

Supplementary Figure S2.

Supplementary Figure S3.

Supplementary Figure S4.

Supplementary Figure S5.

Supplementary Figure S6.

Supplementary Figure S7.

Supplementary Figure S8.

A

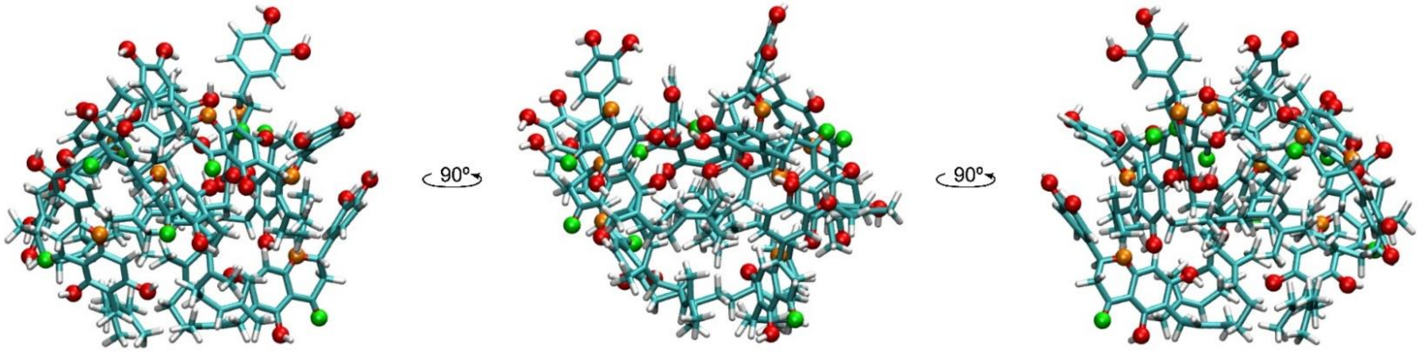

B

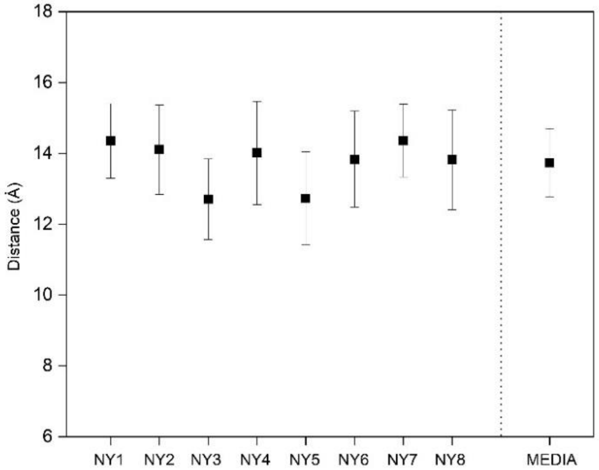

Figure S1. (A) Structure of the aggregate of NYA molecules at the last frame for system 4 (t = 600 ns). NYA molecules (eight) are shown in licorize representation, whereas the oxygens are shown in VDW representation (hydroxyl, carbonyl and ether oxygens in red, green and orange colours, respectively). (B) Average distance between the carbons C4' and the last one of the geranyl chain in the aggregate for each one of the NYA molecules as well as their average. Data correspond to the last 30 ns of molecular dynamics simulation.

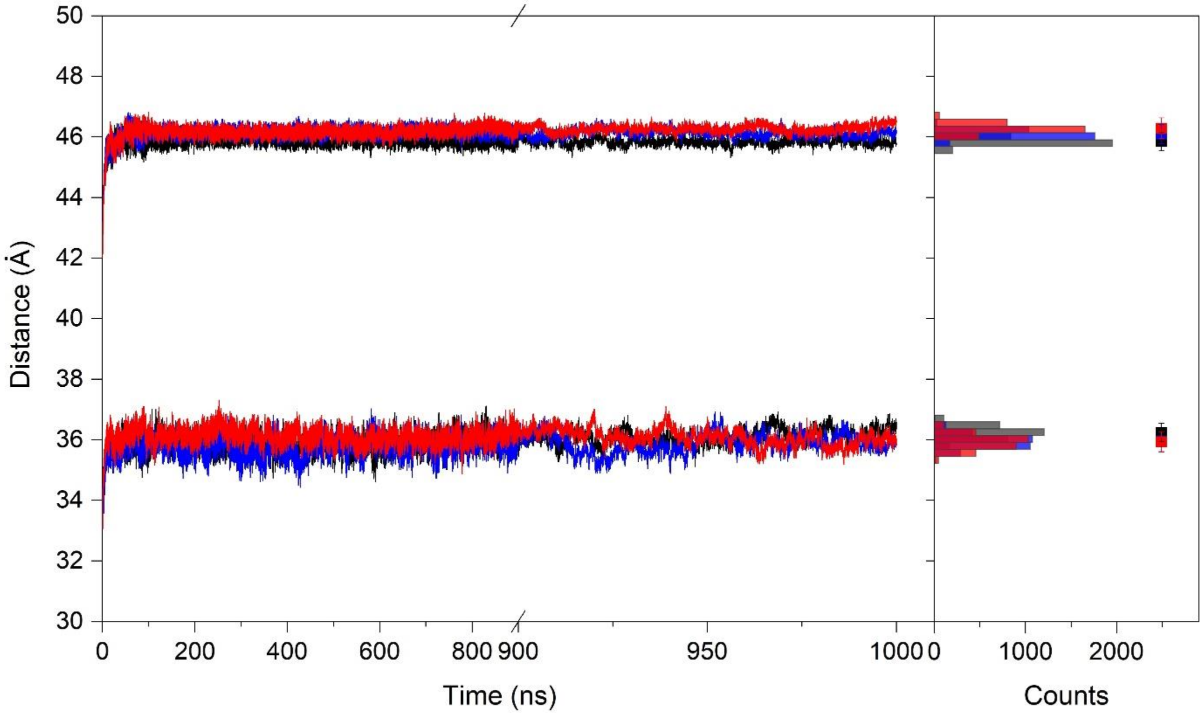

Figure S2. Time variation of membrane thickness for the whole simulation time and the corresponding histograms for the last 30 ns of molecular dynamics simulation. Thickness corresponds to the phosphate atoms of the phospholipids (upper lines) and the oxygen atoms of cholesterol (lower lines) for system 1 (black), system 2 (blue) and system 3 (red).

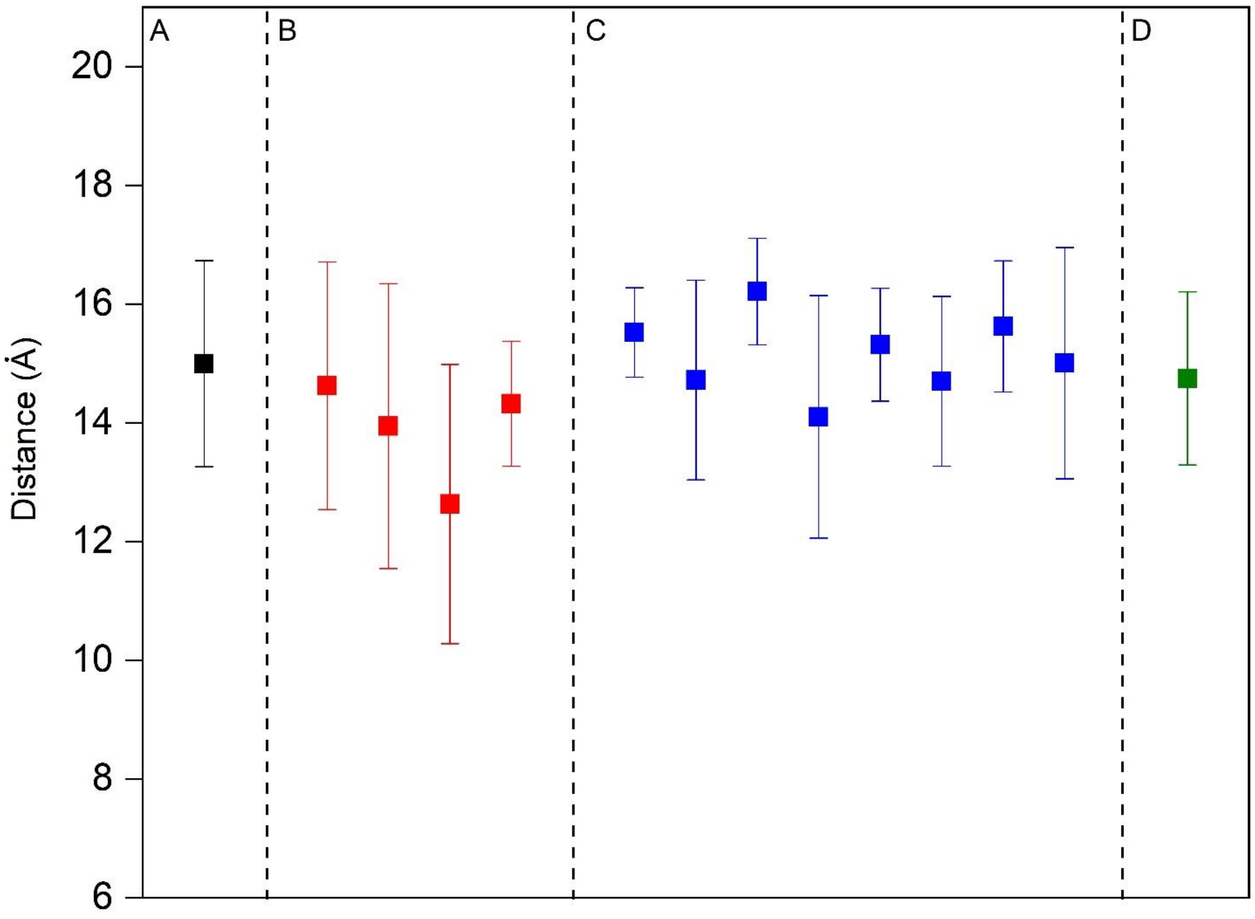

Figure S3. Average distance between carbons C4' and the last one of the geranyl chain for the NYA molecules pertaining to (A) system 1 (one NYA molecule), (B) system 2 (four NYA molecules), and (C) system 3 (eight NYA molecules). In (D) the average ± SD is shown. Data correspond to the last 30 ns of molecular dynamics simulation.

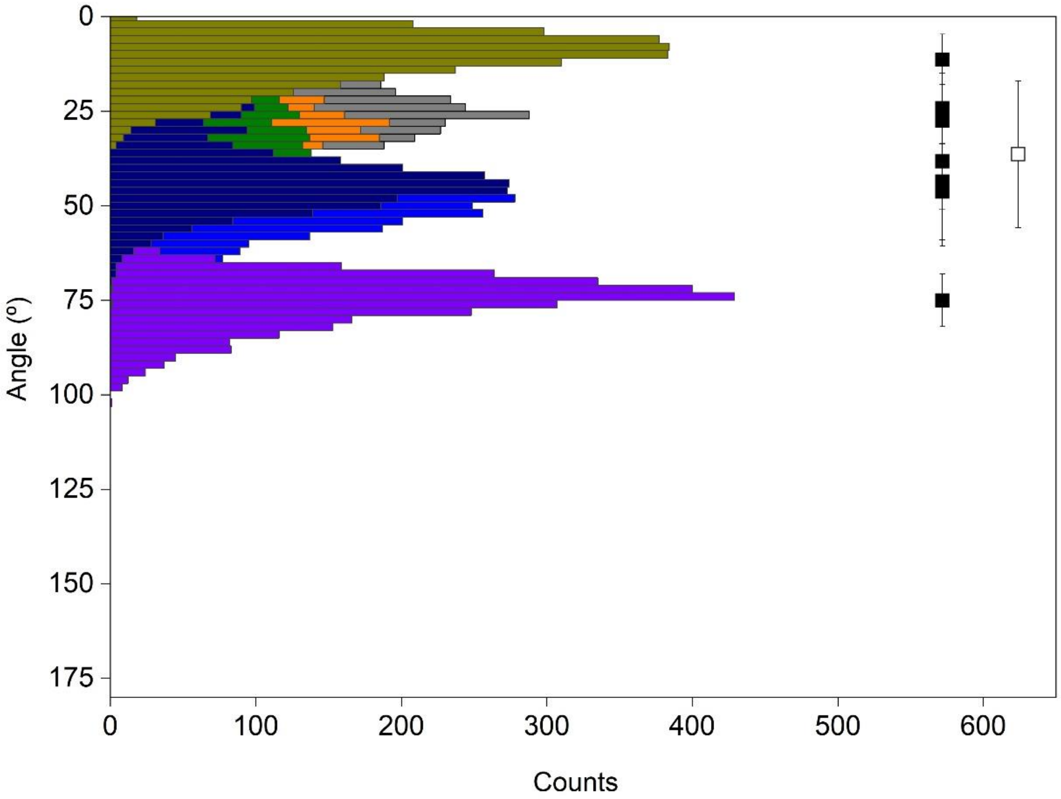

Figure S4. Histograms and average angles (± SD) formed by carbons C4’ and the last one of the geranyl chain for the NYA molecules in monomeric form with respect to the membrane surface. The full symbols correspond to the average angle of each one of the NYA molecules, whereas the empty symbol corresponds to the global average. The data is the average for the last 30 ns of molecular dynamics simulation time.

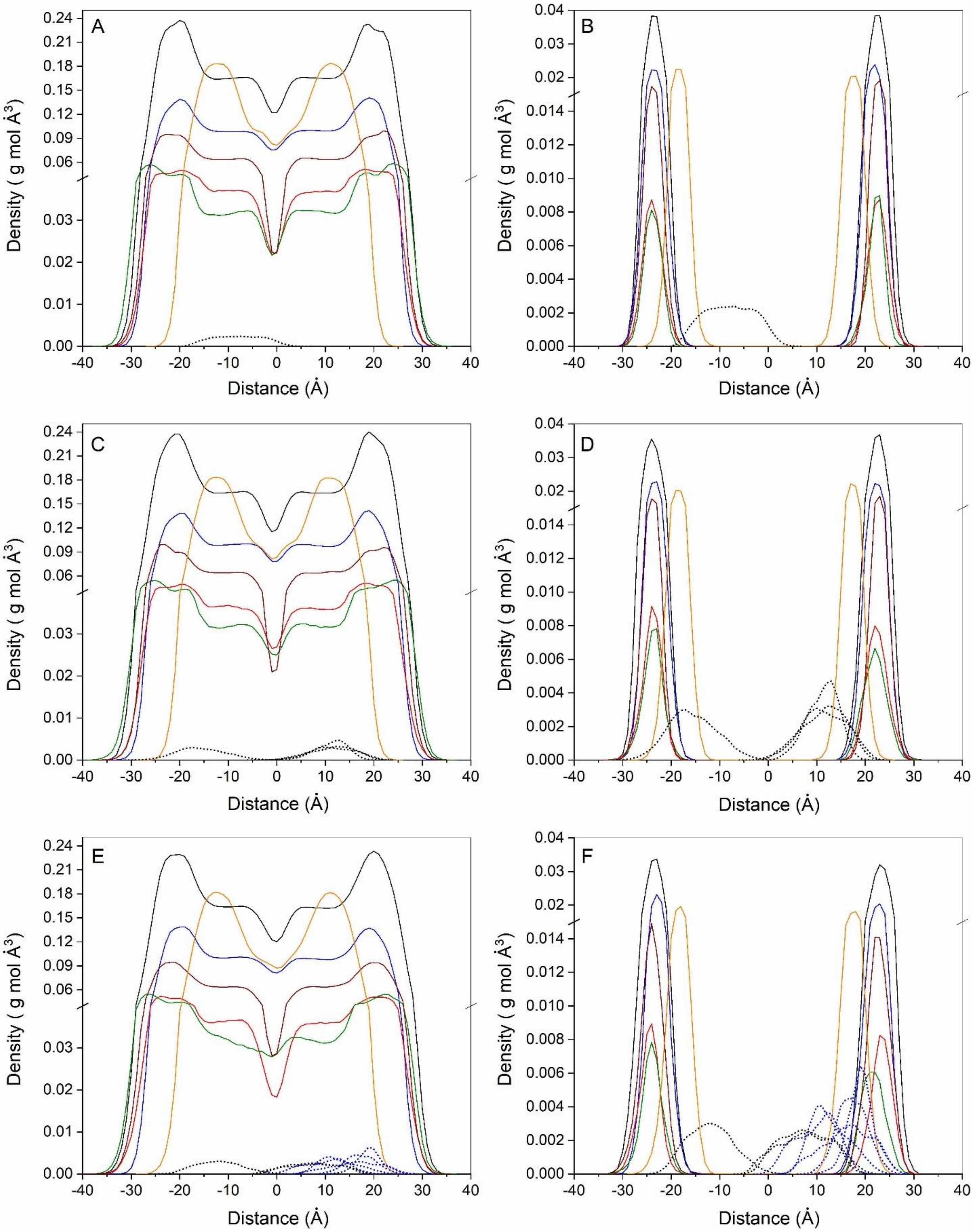

Figure S5. Mass density profiles for the last 30 ns of the molecular dynamics for (A,B) system 1, (C,D) system 2 and (E,F) system 3. (A,C,E) Whole lipid molecules and (B,D,F) phosphorous atoms of phospholipids and the oxygen atoms of CHOL. POPC is shown in black, POPE in blue, POPS in red, PI-3P in olive, PSM in wine and CHOL in orange. The monomeric and aggregated NYA molecules are shown in dotted black and blue colours.

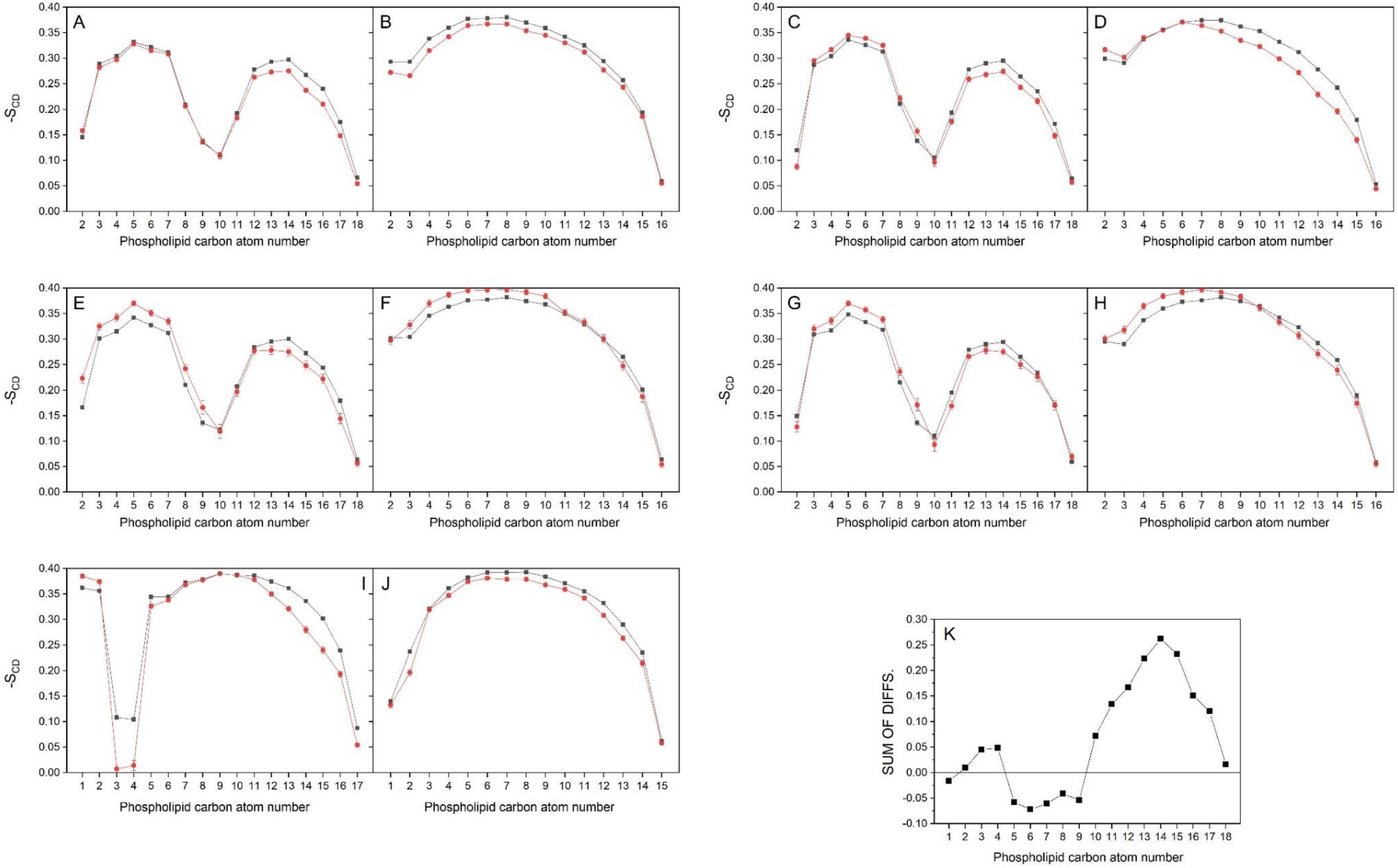
A
B
C
D
E
F
G
H
I
J
K
-S_CD
SUM OF DIFFS.
Phospholipid carbon atom number

Figure S6. Average deuterium order parameter, $-S_{CD}$, calculated for the hydrocarbon chains of the phospholipids. (A,C,E,G) Oleoyl and (B,D,F,H) palmitoyl acyl chains of (A, B) POPC, (C, D) POPE, (E, F) POPS, (G,H) PI-3P, as well as the palmitoyl (I) and sphyngosyl (J) acyl chains of PSM. The data correspond to the bulk phospholipid acyl chains (-■-) and the phospholipid acyl chains within 6 Å of the NYA molecule (-●-). The analysis was carried out for the last 30 ns of simulation. (K) Sum of differences between the $-S_{CD}$ values of the bulk lipids minus the S_{CD} values of the surrounding ones.

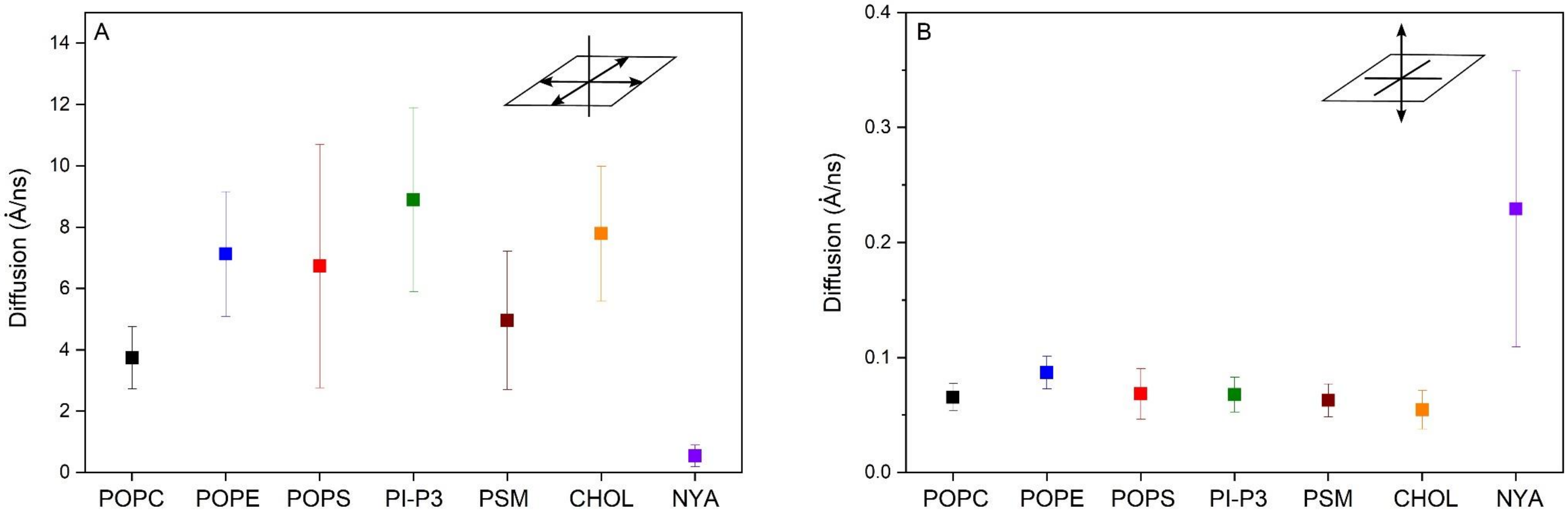

Figure S7. (A) x-y and (B) z diffusion coefficients (Å^2/ns) for the NYA molecules in systems 1,2 and 3 in the monomeric form: POPC (■), POPE (■), POPS (■), PI-3P (■), PSM (■), CHOL (■) and NYA (■). The data is the average for the last 30 ns of molecular dynamics simulation time.

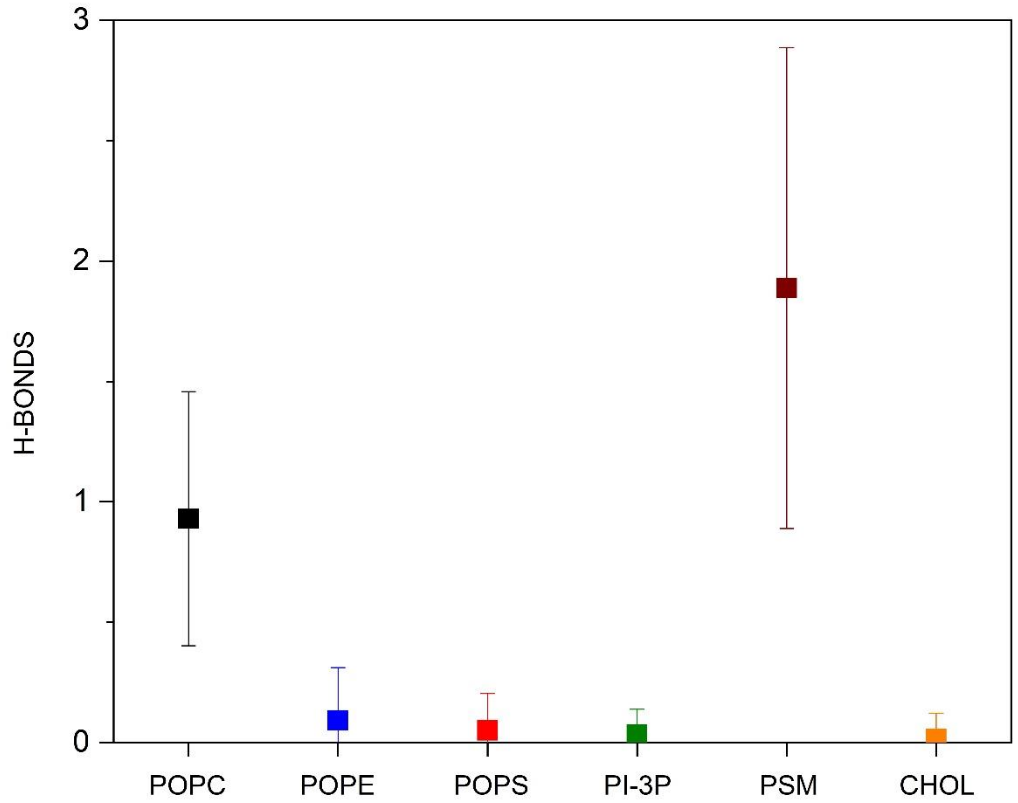

Figure S8. Average number of hydrogen bonds between the membrane lipids and NYA: POPC (-■-), POPE (-■-), POPS (-■-), PI-3P (-■-), PSM (-■-) and CHOL (-■-). The analysis was carried out for the last 30 ns of simulation.
